# Glutamate, NAA, and energy metabolism in clinical high risk and first episode psychosis

**DOI:** 10.1038/s41598-025-22845-y

**Published:** 2025-11-25

**Authors:** Bridget King, Matthew J. Kempton, Shue Kit Man, Alice Egerton, Romina Mizrahi

**Affiliations:** 1 Department of Psychosis Studies, Institute of Psychiatry, Psychology and Neuroscience, King’s College London, London, UK; 2 Douglas Research Centre, Clinical and Translational Sciences Lab, Montreal, QC Canada; 3 Department of Psychiatry, McGill University, Montreal, QC H4H 1R3 Canada

**Keywords:** Glutamate, NAA, Energy metabolism, Mitochondrial function, Clinical high risk, First episode psychosis, Diseases, Neurology, Neuroscience

## Abstract

Regulation of brain glutamate is closely related to brain energy metabolism. Changes in both central glutamatergic function and peripheral energy metabolism have been implicated in psychosis risk, onset and long-term illness, but there is a lack of empirical evidence to link these processes. We investigated the relationships between glutamate and *N*-acetyl-aspartate (NAA, a potential marker of neuronal metabolic integrity) in the anterior cingulate cortex (ACC), measured using proton magnetic resonance spectroscopy (^1^H-MRS), and peripheral markers of energy metabolism (mitochondrial complex I–V content, pyruvate and lactate) in individuals either at clinical high risk for psychosis or in the first episode of psychosis (*N* = 36) and healthy controls (*N* = 20). ACC Glx (glutamate + glutamine) levels were positively related with principal components relating to mitochondrial complex content, and this relationship did not differ between groups. These findings are consistent with the importance of mitochondrial ATP generation in regulating glutamatergic neurotransmission. While we did not find evidence that this relationship is disrupted in clinical high risk or first episode psychosis, further work is required to understand the mechanisms linking glutamate and energy metabolism in psychosis, including studies in larger cohorts, later stages of illness or in individuals with greater illness burden.

**Supplementary Information:**

The online version contains supplementary material available at 10.1038/s41598-025-22845-y.

## Introduction

The psychosis clinical high risk (CHR) state is defined as an early disease stage prior to the onset of overt psychosis and is characterised by sub-threshold psychotic or non-specific psychiatric symptoms^1^. Individuals meeting CHR criteria have a 25% probability of transitioning to first episode psychosis (FEP) within 3 years, and a 35% probability within 10 years^2^. Understanding the active biological mechanisms at the early stages of psychosis is crucial for elucidating the pathophysiology underlying psychosis risk and onset, and for identifying potential targets for early intervention.

Recent reviews have highlighted the potential importance of the interplay between regulation of brain glutamate, mitochondrial dysfunction and energy metabolism in psychosis pathophysiology and onset^3–5^. Glutamatergic neurotransmission has been extensively associated with schizophrenia pathophysiology^6,7^. Maintaining ion gradients to support glutamatergic signalling and action potentials is the most energy-intensive process in the brain, consuming the majority of ATP produced via cortical glucose metabolism and accounting for an estimated 75–80% of total glucose use^8–10^. In addition to the energy requirements of glutamate homeostasis through the glutamate-glutamine cycle^11,12^, high concentrations of glutamate can inhibit mitochondrial complexes^13–15^ thus disrupting ATP production which may increase neuronal vulnerability to excitotoxicity^16^. In response, lactate and pyruvate levels may rise, serving as alternative energy substrates and neuroprotective factors by sustaining ATP production and buffering against metabolic stress^17,18^.

It has been proposed that during the early stages of psychosis, genetic and environmental risks converge to impair ATP production from glucose through oxidative phosphorylation in mitochondria, which may progress to compensatory increases in aerobic glycolysis and lactate formation at later illness stages^4,5,19^. However, most of the empirical evidence for mitochondrial dysfunction and disrupted energy metabolism in psychosis is derived from studies in chronic schizophrenia, (including postmortem), with relatively little in vivo investigation of the active mechanisms at the early stages of psychosis.

Glutamate can be measured in vivo using proton magnetic resonance spectroscopy (^1^H-MRS). ^1^H-MRS meta-analyses have shown that compared to healthy volunteers, individuals with psychosis-related conditions, including schizophrenia, show an overall reduction in glutamate in the medial frontal cortex (mFC), including the anterior cingulate cortex (ACC)^20–22^. However, mFC/ACC glutamate levels may be increased at earlier illness stages, including in CHR individuals^23^ or FEP^20,22^. Moreover, psychosis is associated with greater variability in MFC glutamate, and this is more pronounced in younger individuals^21^. This may suggest greater disturbances in energy-expensive glutamate regulatory mechanisms at earlier illness stages.

^1^H-MRS also provides measurement of N-acetyl aspartate (NAA), which is primarily synthesised in neuronal mitochondria^24^ and may provide a marker of neuronal metabolic integrity^25,26^ and mitochondrial energy output^27^. NAA levels in the frontal lobe are reduced in CHR^28,29^, FEP and chronic schizophrenia^30^. Together, these studies therefore indicate increased and more variable levels of MFC glutamate and decreased NAA, which may indicate impaired neuronal mitochondrial activity, during the clinical high risk and early stages of psychosis.

In early psychosis, elevations in peripheral markers of mitochondrial dysfunction are associated with greater symptom severity, poorer functioning and neurocognitive abilities^31^. Mitochondrial complex I activity may be elevated^32^ and positively correlated with positive and cognitive symptom severity^33^. In CHR individuals, while mitochondrial complex content does not differ compared to in healthy controls, complex III content appears negatively associated with prodromal negative and total symptom severity scores, and complex V content positively correlated with disorganisation severity scores^19,34^. Although brain lactate has not yet been investigated in CHR or FEP individuals, analysis of peripheral lactate and pyruvate suggests no change^19^ or decreases during the CHR stage^35,36^ but increases during FEP^37^. However, the relationships between brain glutamate and NAA with peripheral markers of energy metabolism have not yet been investigated.

In this study we tested the hypothesis that in CHR and FEP individuals compared to healthy controls, brain glutamate, measured using ^1^H-MRS, would be negatively associated with peripheral mitochondrial complex content and positively associated with lactate and pyruvate levels; and that NAA, as a marker of metabolic integrity, would show the opposite relationships. In exploratory analyses we tested for associations between these markers, symptom severity and cognitive performance.

## Methods

### Regulatory approvals

The study was performed in collaboration with Clinical and Translational Sciences CaTS BioBank under a repository protocol that allowed a re-analysis of previously acquired data approved by the Centre for Addiction and Mental Health Research Ethics Board and now approved under Clinical and Translational Sciences (CaTS) BioBank by the Research Ethics Board (REB) of the Centre Intégré Universitaire de Santé et de Services Sociaux (CIUSSS) de l’Ouest-de-l’Île-de-Montréal—Mental Health and Neuroscience subcommittee for secondary analyses.

Participants included in the study were subset of a larger dataset recruited from July 20, 2011, to March 12, 2019 (Ontario, Canada). The study was performed in accordance with Good Clinical Practice guidelines, regulatory requirements, and the Code of Ethics of the World Medical Association (Declaration of Helsinki). Written informed consent was obtained from all participants after a full explanation of study procedures. All CHR and FEP individuals had capacity to provide informed consent, as assessed by MacArthur Competence Assessment Tool for Clinical Research (MacCAT).

The dataset reported here partially overlaps with previously published cohorts, including 1 H-MRS ACC^38^, mitochondrial complex analyses^19^ and C4A expression^39^. The analytic sample comprised 56 participants: 26 at CHR, 10 with FEP, and 20 healthy controls (HCs). HCs included in this study did not meet the diagnostic criteria for cannabis use disorder^38^.

To maximise statistical power and availability of both ^1^H-MRS and peripheral energy measures, CHR and FEP participants were combined into a single CHR + FEP group. Participants in the CHR + FEP and HC groups were then matched using propensity score matching (via the *MatchIt* package in R), based on age and sex.

### Participants and clinical assessments

CHR participants met the diagnostic criteria for prodromal risk syndrome which was assessed using the criteria of prodromal syndromes (COPS)^40^. CHR participants were excluded if they currently met the criteria for any Axis I disorder, assessed by the structured clinical interview for DSM-IV (SCID-I)^41^. The severity of prodromal symptoms in the CHR group was assessed using the structured interview for prodromal syndromes (SIPS) and scale of prodromal symptoms (SOPS)^40^. The SOPS scores consist of four categories: positive, negative, general and disorganised symptoms^40^. FEP participants met the diagnostic criteria of schizophrenia, schizophreniform disorder, delusional disorder or psychosis, as determined by the SCID-I^41^ and were within 36 months of their initial diagnosis. In FEP participants, symptom severity was assessed using the Positive and Negative Syndrome Scale (PANSS)^42^. FEP participants were excluded if their psychotic symptoms were better explained by bipolar disorder or another concurrent DSM-IV Axis I Disorder. HCs were defined as having no history of psychiatric illness or first degree relative with a psychotic disorder, determined by the SCID-I^41^. Exclusion criteria for all participants included pregnancy or breastfeeding, meeting criteria for alcohol and/or substance abuse disorder, and standard contraindications to MRI. Functioning was assessed using the Global Assessment of Functioning scale (GAF)^43^. Neurocognitive performance was assessed using the Wisconsin Card Sorting Test (WCST). All participants underwent urine drug screens for recreational substances at the time of MRI and blood sample collection.

### Symptom severity scores

Since the positive items in the SOPS are derived directly from the positive PANSS scale^40^, we calculated a combined positive score for the CHR and FEP groups, following the approach described previously^44^.

### Proton magnetic resonance spectroscopy

As previously described^38^, ^1^H-MRS scans were performed at the CAMH Research Imaging Centre in Toronto, Canada with a 3 Tesla General Electric scanner and 8-channel head coil. To minimise head motion, participants were positioned with a soft restraint padding placed around the head and with tape strapped across the forehead. T1-weighted fast spoiled-gradient-echo 3-dimensional sagittal acquisition scans were acquired for each participant (FSPGR sequence, TE = 3.0 ms, TR = 6.7 ms, TI = 650 ms, flip angle = 8°, FOV = 28 cm, acquisition matrix 256 × 256 matrix, slice thickness = 0.9 mm).

The ^1^H-MRS voxel was positioned in the bilaterial supragenual anterior cingulate cortex (ACC, 30 × 20 × 15 mm). ^1^H-MRS spectra were acquired using the standard GE Proton Brain Examination (PROBE) sequence using PRESS (Point Resolved Spectroscopy Sequence), at TE = 35 ms, TR = 2000 ms, number of excitations = 8, bandwidth = 5000 Hz, 4096 data points, 128 water-suppressed, and 16 water-unsuppressed averages. The target water linewidths after shimming were 12 Hz or less. Voxel placement and example spectra are shown in Supplementary Fig. 1.

Spectra were analysed using LCModel Version 6.3–1N^45^. Pre-processing included eddy-current correction and water-scaling. A standard LCModel basis set for PRESS at 3T (TE = 35 ms), provided by^45^ was used. This basis set includes simulated spectra for glutamate (Glu), glutamine (Gln), combined glutamate–glutamine (Glx), N-acetylaspartate (NAA), myo-inositol (mI), choline-containing compounds (Cho), γ-aminobutyric acid (GABA), and glutathione (GSH). Gannet 2.0 software (version 2.0, http://gabamrs.com/) co-registered the ^1^H-MRS voxel onto the corresponding segmented T1-weighted image to extract the grey matter, white matter and cerebrospinal fluid in the voxel^46^. Metabolite values were corrected using the voxel tissue contents using the formula: M*corr* = M * (WM + 1.21 * GM + 1.55 * CSF)/(WM + GM) whereby M = uncorrected metabolite and WM, GM and CSF indicating the white and grey matter and cerebrospinal fluid content. This equation assumes a CSF concentration of 55.556 mol/L^47,48^.

^1^H-MRS spectral quality was determined by review of LCModel estimates of spectral full-width-half-maximum (FWHM) and signal-to-noise (SNR) ratio. Predefined criteria for data exclusion were spectra associated with a FWHM or SNR 2 standard deviations respectively above or below the mean values for the dataset, and individual metabolite concentration estimates associated with a Cramer Rao Lower Bounds (CRLB) > 20%. No data were excluded based on these quality criteria.

The primary ^1^H-MRS outcome variables were glutamate, Glx and NAA. Due to overlapping resonances at 3 Tesla, NAA is reported as the sum of N-acetyl aspartate (NAA) and N-acetyl aspartyl glutamate (NAAG).

### Peripheral energy measures

Mitochondrial complex I–V protein content in monocyte samples was measured using a multiplex ELISA assay, as previously described^19^. Protein levels were expressed as a percentage relative to nicotinamide nucleotide transhydrogenase (%NNT), to account for potential differences in mitochondrial DNA content between individuals^34^. Plasma lactate and pyruvate were measured using colorimetric L-Lactate and Pyruvate Assay Kits, also as previously described^19^, and are reported in nmol/µL.

### Statistical analysis

Data were analysed using RStudio Version 2024.4.2.764^49^, using the packages tidyverse^50^, ppcor^51^, car^52^, boot^53^, and Hmisc^54^. Figures were made using ggplot2^55^.

To address multicollinearity among mitochondrial complexes I–V, a principal component analysis (PCA) was conducted. Variables were standardised prior to analysis by conversion to Z scores. The number of components retained was determined based on inspection of the scree plot and the eigenvalue > 1 criterion (presented in supplementary materials). Component scores from the retained components were extracted and used in subsequent analyses.

Initial analyses tested for group differences in demographic and clinical characteristics, ^1^H-MRS metabolites and peripheral energy measures using T-tests for continuous variables and Chi-Squared Tests for categorical variables, or for non-parametric data Mann–Whitney U Tests and Fisher’s Exact Test respectively. General linear models tested the association between ^1^H-MRS metabolites and peripheral energy measures across the whole sample, including a group by ^1^H-MRS metabolite interaction term to test for group differences. Non-parametric data were analysed using Mann-Whitney U tests for group differences and bootstrapped general linear models for associations between ^1^H-MRS metabolites and peripheral energy measures, including a group by ^1^H-MRS metabolite interaction term to test for group differences.

Follow up analyses controlled for group differences in tobacco use with ANCOVAs and additional general linear models, as appropriate. In exploratory analyses, bivariate correlations tested for associations between ^1^H-MRS metabolites and peripheral energy markers with symptom severity and cognitive performance.

Statistical significance was defined as *P* < 0.05 for associations between ^1^H-MRS metabolites and peripheral energy measures. Benjamini–Hochberg false discovery rate (FDR) using a Q threshold of 10% was applied to analyses of associations between ^1^H-MRS metabolites and peripheral energy measures with symptom severity and cognitive performance to control for multiple comparisons.

## Results

### Participant characteristics

Clinical and demographic information is provided in Table 1. The dataset for analysis comprised of 36 CHR + FEP participants and 20 HCs. Peripheral lactate and pyruvate levels were available 29 CHR + FEP participants and 14 HCs. In the CHR + FEP group, three individuals were receiving risperidone, and one was receiving quetiapine. In addition, four participants (two CHR, two FEP) were receiving antidepressants, two participants (one CHR, one FEP) were receiving benzodiazepines, and one CHR participant was receiving lisdexamfetamine. Four participants in the CHR + FEP group and 2 HCs had a positive drug screen for cannabis.

Compared to HCs, the CHR + FEP group had significantly higher tobacco use and lower GAF scores. There were no significant differences between the CHR + FEP group and HCs in the remaining demographic characteristics and cognitive scales (Table 1).

**Table 1:** Clinical and demographic Information.

	CHR + FEP (*N* = 36)	HCs (*N* = 20)	*P* value
CHR/FEP: n group	26/10	–	–
Sex (M/F)	22/14	7/13	*P* = 0.111
Age:	21.64 (3.62)	21.15 (1.87)	*P* = 0.576
BMI	24.03 (5.75)	23.73 (5.21)	*P* = 0.855
Positive urine for cannabis (Y/N)	4/32	2/18	*P* = 1.000
Cumulative cannabis exposure	467.76 (191.46)	168.21 (475.56)	*P* = 0.239
Tobacco (Y/N)	10/26	0/20	*P* = 0.009*
Current antipsychotic use in FEP (Y/N)	4/6	–	–
Psychotropic use: Es/Lo/Lis/Se/Ve	2/2/1/1/1	–	–
Symptoms and functioning:			
Positive symptom severity	13.70 (5.21)	–	–
GAF	50.92 (8.08)	83.4 (5.95)	*P* < 0.001*
Cognitive function (WCST):			
N data points available	30	17	
Correct mean categories	19.39 (4.45)	18.40 (3.02)	*P* = 0.420
Perseverative errors	2.69 (2.48)	2.12 (0.88)	*P* = 0.364

Values are presented as Mean ± SD. The CHR + FEP group comprised individuals meeting criteria for clinical high risk (CHR) of psychosis or first episode psychosis (FEP). Psychotropic medications: Es, escitalopram; Lis, lisdexamfetamine; Lo, lorazepam; Se, sertraline; Ve, venlafaxine; BMI, body mass index; GAF, global assessment of functioning; WCST, Wisconsin card sorting test. *Denotes significance at *P* < 0.05.

### Group differences in ^1^H-MRS metabolites and energy measures

Of the 56 participants, 55 had complete data across complexes I–V and were included in the PCA; one participant was excluded due to missing data. The PCA for mitochondrial complexes I–V identified two principal components with eigenvalues greater than 1. PC1 explained 48.51% of the variance and showed strong positive loadings from complexes I, II, and IV. PC2 explained 28.50% of the variance, with a strong positive loading from complex V and a strong negative loading from complex III (presented in supplementary materials).

There were no significant group differences in glutamate, Glx, NAA, mitochondrial complex content (PC1 and PC2), lactate, pyruvate or lactate/pyruvate (LP) ratio between the CHR + FEP group and HCs (Table 2), which remained unchanged after covarying for group differences in SNR (presented in supplementary materials). After covarying for group differences in tobacco use, NAA (F (1,53) = 4.254, *P* = 0.044) and pyruvate (F (1,40) = 4.30, *P* = 0.045) were higher in the CHR + FEP group compared to HCs.

**Table 2:** 1 H-MRS data quality, voxel tissue contents, metabolites, energy measures.

	CHR + FEP (*N* = 36)	HCs (*N* = 20)	T or U statistic	df	*P* value
^1^H-MRS data quality					
SNR	27.00 (5)	30.00 (5)	T = − 2.25	54	*P* = 0.028*
FWHM	0.03 (0.01)	0.03 (0.01)	T = 1.62	54	*P* = 0.110
Voxel tissue contents					
GM	0.70 (0.04)	0.70 (0.04)	T = − 0.11	54	*P* = 0.913
WM	0.16 (0.03)	0.15 (0.03)	T = 0.29	54	*P* = 0.773
CSF	0.16 (0.03)	0.16 (0.04)	T = − 0.11	54	*P* = 0.913
^1^H-MRS metabolites in the ACC					
Glutamate _corr_	16.65 (1.54)	16.38 (1.36)	T = 0.67	54	*P* = 0.514
Glx _corr_	21.84 (2.53)	21.69 (2.67)	T = 0.22	54	*P* = 0.830
NAA _corr_	15.16 (0.88)	15.02 (0.87)	T = 0.59	54	*P* = 0.558
Peripheral markers of energy metabolism					
Mitochondrial complex PC1	0.27 (1.45)	− 0.51(1.69)	T = 1.78	53	*P* = 0.080
Mitochondrial complex PC2	− 0.09 (1.25)	0.18 (1.08)	T = − 0.80	53	*P* = 0.430
Pyruvate^†,#^	0.08 (0.04)	0.07 (0.03)	U = 234	–	*P* = 0.415
Lactate^#^	2.78 (0.84)	2.71 (0.99)	T = 0.20	41	*P* = 0.842
LP ratio	38.56 (10.12)	42.87 (11.52)	T = 1.25	41	*P* = 0.218

^1^H-MRS metabolite values are corrected for voxel tissue content and expressed as Mean (SD). ^†^Indicates that the variable did not meet parametric assumptions and is expressed as the Median (interquartile range). # Indicates that data available for 29 CHR + FEP and 14 HC. Group differences in parametric variables were tested using independent samples t-tests and non-parametric variables were tested using Mann–Whitney U tests. T statistics are reported for parametric variables and U statistics are reported for non-parametric variables.

CSF, voxel cerebrospinal fluid fraction; FWHM, full width at half maximum; Glu, glutamate; Glx, glutamate + glutamine; GM, voxel grey matter fraction; LP ratio, lactate-to-pyruvate ratio; NAA, *N*-acetylaspartate plus *N*-acetylaspartyl glutamate; SNR, signal to noise ratio; WM, voxel white matter fraction. *Denotes significance at *P* < 0.05.

### Relationships between ^1^H-MRS metabolites and peripheral energy measures

**Fig. 1:**
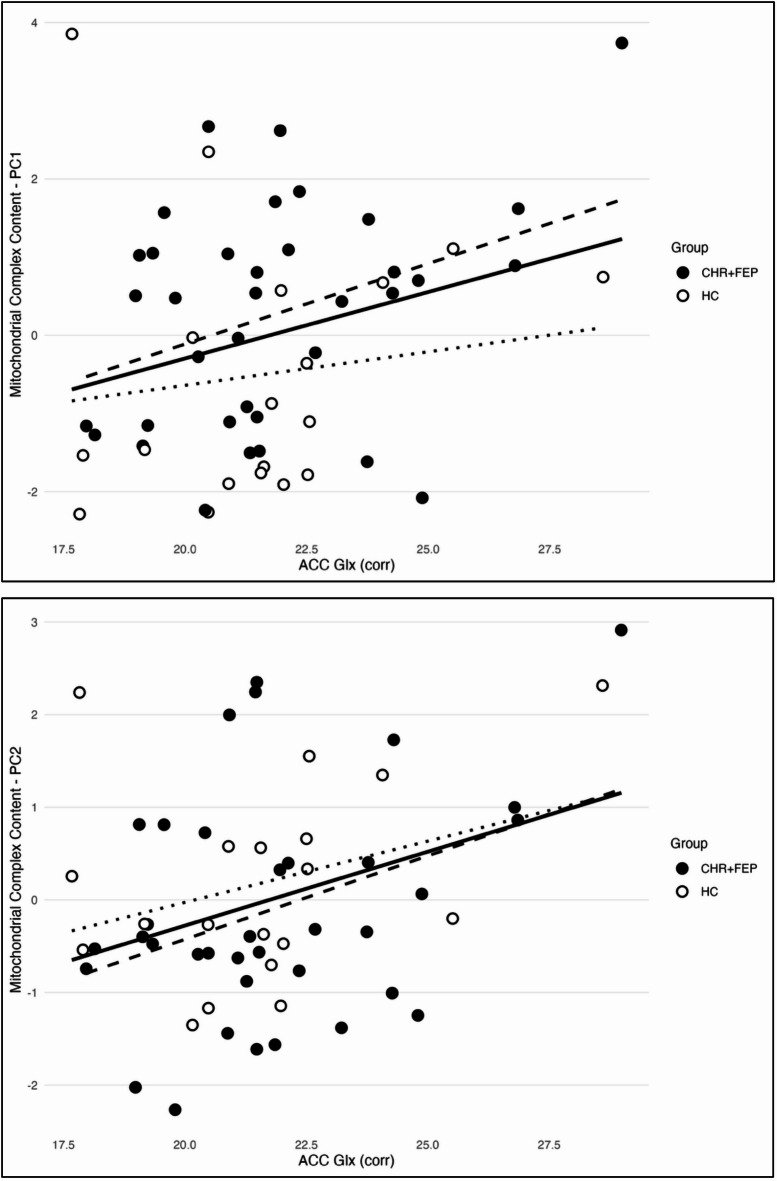
Associations between Glx_corr_ levels in the anterior cingulate cortex (ACC) and mitochondrial complex content PC1 (Estimate = 0.632, T = 2.155, *P* = 0.036) and PC2 (Estimate = 0.734, T = 2.22, *P* = 0.031). The solid black line represents the line of best fit for the total sample. The dashed line represents the fit for the CHR + FEP group and the dotted line represents the fit for the HC group. Data points are plotted by group, with solid circles indicating CHR + FEP participants and unfilled circles indicating HC participants.

Across the whole sample, ACC Glx was positively associated with both mitochondrial complex content PC1 (Estimate = 0.632, *T* = 2.16, *P* = 0.036) and PC2 (Estimate = 0.734, *T* = 2.22, *P* = 0.03) (Fig. 1). These associations remained significant after controlling for tobacco use (PC1: Estimate = 0.666, *T* = 2.24, *P* = 0.029; PC2: Estimate = 0.753, *T* = 2.23, *P* = 0.029). Critically, there were no significant group by PC1 (Estimate = -0.419, *T* = − 0.920, *P* = 0.363) or group by PC2 (Estimate = 0.061, *T* = 0.10, *P* = 0.923) interaction effects.

Exploratory post hoc analysis indicated that Complex I (rho (56) = 0.329, *P* = 0.013) and Complex V (rho (56) = 0.346, *P* = 0.009) content individually correlated with Glx, indicating that these complexes may contribute most strongly to the associations of PC1 and PC2 respectively with Glx.

There were no further significant overall associations between ^1^H-MRS metabolites and peripheral energy measures, or group by metabolite interactions, including when covarying for group differences in tobacco use (see supplementary material).

### Relationships with cognition and symptoms

There were no significant correlations between ^1^H-MRS metabolites or peripheral energy-related measures and the mean number of categories or perseverative errors on the WCST across the whole sample (presented in supplementary materials). In the CHR + FEP group, there were no significant associations between combined positive symptom severity scores and ^1^H-MRS metabolites or peripheral energy-related measures (presented in supplementary materials).

## Discussion

The aim of this study was to investigate the relationships between anterior cingulate cortex (ACC) glutamate and N-acetylaspartate (NAA) levels and peripheral markers of energy-metabolism in individuals at clinical high risk of psychosis (CHR) or in the first episode of psychosis (FEP) and healthy controls (HCs). Contrary to our hypotheses, that ACC glutamate metabolites would be negatively associated with peripheral mitochondrial complex content in the CHR + FEP group, we found significant positive associations between Glx (glutamate + glutamine) levels with principal components relating to mitochondrial complex content, which did not differ between groups. NAA, which may provide a marker of metabolic integrity in the ACC, was not significantly associated with peripheral energy measures.

As prior evidence overall indicates a decrease in mitochondrial activity^56^ and an increase in ACC glutamate metabolites^20,23^ in the clinical high risk or early stages of psychosis, we hypothesised that these markers would be negatively correlated. However, studies in both CHR and FEP cohorts have revealed mixed findings regarding the direction of change in ACC glutamate metabolites, and it is also possible that glutamate levels differ between these groups. For example, studies of CHR participants have reported both elevated ACC glutamate^57^ and no differences relative to healthy controls^58^, and some studies in FEP, particularly those at 7T, have tended to show lower glutamate compared with controls^59–62^. The complexity of these findings may reflect clinical and glutamatergic differences between individual participants or cohorts, ^1^H-MRS methodology including ability to resolve glutamate from glutamine amongst other factors. The limited robustness of a glutamate signal change in CHR or FEP participants may explain the lack of significant difference in glutamate metabolites in the CHR + FEP compared to control group, or a correlation between glutamate and markers of energy metabolism in the CHR + FEP group alone.

Contrary to our hypotheses, we found a significant positive relationship across all participants (CHR, FEP and HCs) between ACC Glx and PC1, reflecting increased content of mitochondrial complexes I, II and IV and Glx and PC2, reflecting the balance between Complex V and III content. Follow-up analysis suggested that positive relationships between content of Complex I (NADH: ubiquinone oxidoreductase) and Complex V (ATP synthase) with ACC Glx may contribute most to these relationships, representing the first and last steps of the mitochondrial respiratory chain. There were no detectable differences in ACC glutamate metabolites or mitochondrial complex content PCs in our CHR + FEP sample compared to HCs, or in the relationship between the mitochondrial PCs and Glx. Our results therefore indicate that peripheral mitochondrial complex content is mainly positively associated with ACC Glx in the absence of marked dysregulation of either marker. This positive relationship is broadly consistent with mechanistic associations between glutamate homeostasis and cellular energy metabolism^12,63^. It may be that disrupted relationships between peripheral markers of mitochondrial complex content and brain glutamate become more apparent during later stages of psychosis/schizophrenia and may differ in studies examining cohorts with greater average illness burden.

In contrast to the positive associations between ACC Glx and mitochondrial complex PCs, we did not detect associations between ACC glutamatergic metabolites or NAA with peripheral lactate or pyruvate levels. This contrasts with a recent finding of a positive correlation between ACC Glx and NAA and peripheral lactate across patients with schizophrenia and HCs^64^. It is possible that this may be due to the more limited sample size of our study, in which both lactate and ACC glutamate metabolites were available in a total of 43 participants, compared to 96 participants in^64^, although our data indicated a non-significant negative relationship. It is also possible that relationships between peripheral lactate and ACC glutamate or NAA only become apparent at later illness stages. According to a recent review^5^, shifts in metabolic processes may occur in chronic stages of schizophrenia following excessive glutamate signalling and redox dysregulation within early illness stages, which “burn out” in chronic schizophrenia, leading to a shift from oxidative phosphorylation to glycolysis. Shifts in metabolic processes occurring in chronic stages of illness aligns with the evidence showing increases in lactate in chronic schizophrenia^65,66^ but not in CHR or FEP, compared to HCs^19^,^67^ as well as increases in the lactate to pyruvate ratio in post-mortem studies^68,69^. Future work should examine associations between lactate and glutamate metabolites in psychosis directly within the same brain region, using lactate-optimised ^1^H-MRS.

In exploratory analysis, we tested whether peripheral energy measures and ^1^H-MRS metabolites were associated with symptom severity and cognitive function and found no significant associations. In early psychosis, greater mitochondrial dysfunction is associated with higher positive symptom burden and worse cognitive functioning^31^, however our study mainly included CHR participants and utilised a different set of mitochondrial markers. Some studies have detected relationships between performance on the WCST in established psychosis and glutamate^70,71^ or NAA^72,73^. However, due to null or opposite findings and methodological heterogeneity, the overall relationships between ^1^H-MRS glutamate or NAA and cognition are not well understood^74^.

### Strengths and limitations

Strengths of our study include the use of ex-vivo measurements of mitochondrial complex content in peripheral blood cells. Most of our sample were unmedicated, which minimised the potential impact of antipsychotic medication on glutamate and NAA^75–79^ as well as peripheral energy measures^80,81^.

Limitations of our study include the relatively small sample size, and we may have lacked power to detect further associations or group differences in associations between ^1^H-MRS metabolites and peripheral energy measures. A retrospective power analysis based on our dataset indicated that a sample size of 159 participants would be required to detect a small interaction effect (f² = 0.05) between metabolite levels and group, at a significance level of 0.05 (two-tailed) with 80% power. To maximise the available data, we combined CHR and FEP participants into one group for analysis, but our ^1^H-MRS and peripheral measures, or their relationships, may change after the onset of psychosis compared to in the clinical high-risk stage. ^1^H-MRS is limited in that it measures the total amount of MR-visible glutamate and glutamine in the voxel, whereas neuronal glutamate and glutamine are more tightly and directly coupled to mitochondrial ATP production^63^. In addition, our assay measured protein content rather than direct enzymatic activity; therefore, results should be interpreted as a proxy for mitochondrial function. Finally, we measured mitochondrial content, pyruvate and lactate in peripheral samples, while glutamate and NAA were assessed in the brain using ^1^H-MRS. The strength of the association between peripheral measurements of energy metabolism and brain energy metabolism are largely unknown, although previous studies in Parkinson’s disease have shown that alterations in mitochondrial complex I activity are the same in the periphery and in the brain^82,83^.

## Conclusion

In conclusion, this study found a significant positive relationship between ACC Glx and principal components relating to peripheral mitochondrial complex content, which did not differ between CHR + FEP participants compared to HCs, and was potentially driven by complex I and V. Future studies in larger samples might investigate whether differential relationships between ^1^H-MRS metabolites and peripheral or central energy measures evolve over the course of psychosis / schizophrenia, potentially in relation to illness burden.

## Supplementary Information

Below is the link to the electronic supplementary material.

Supplementary Material 1

## Data Availability

The datasets generated and/or analysed during the current study are available from the corresponding authors upon reasonable request.
