# Supplementary material for: Glutamate, NAA, and energy metabolism in clinical high risk and first episode psychosis
Source: Sci Rep. 2025 Nov 25;15:42031. doi: 10.1038/s41598-025-22845-y (PMC12658253; doi:10.1038/s41598-025-22845-y)
Supplement: Supplementary file 1 — Supplementary Material 1 [file 41598_2025_22845_MOESM1_ESM.docx]

**Supplementary Materials: Glutamate, NAA, and Energy Metabolism in Clinical High Risk and First Episode Psychosis**

1. **Supplementary Figure 1.** Voxel placement in the ACC with example spectrum
2. **Supplementary Figure 2.** Principal Component Analysis **–** Scree Plot
3. **Supplementary Table 1.** Factor loadings of principal components
4. **Supplementary Table 2:** Group differences in ^1^H-MRS metabolites and peripheral energy measures between CHR+FEP and HC, covarying for signal-to-noise-ratio
5. **Supplementary Table 3.** Group differences in ^1^H-MRS metabolites and peripheral energy measures between CHR+FEP and HC, covarying for tobacco use
6. **Supplementary Table 4.** Associations between ^1^H-MRS metabolites and peripheral energy measures
7. **Supplementary Table 5.** General linear models tested associations between ^1^H-MRS metabolites and peripheral energy measures, covarying for tobacco use
8. **Supplementary Table 6.**Associations between ^1^H-MRS metabolites and peripheral energy measures with the number of categories on the WCST
9. **Supplementary Table 7.**Associations between ^1^H-MRS metabolites and peripheral energy measures with the number of perseverative errors on the WCST
10. **Supplementary Table 8.**Associations between ^1^H-MRS metabolites and peripheral energy measures with positive symptom severity


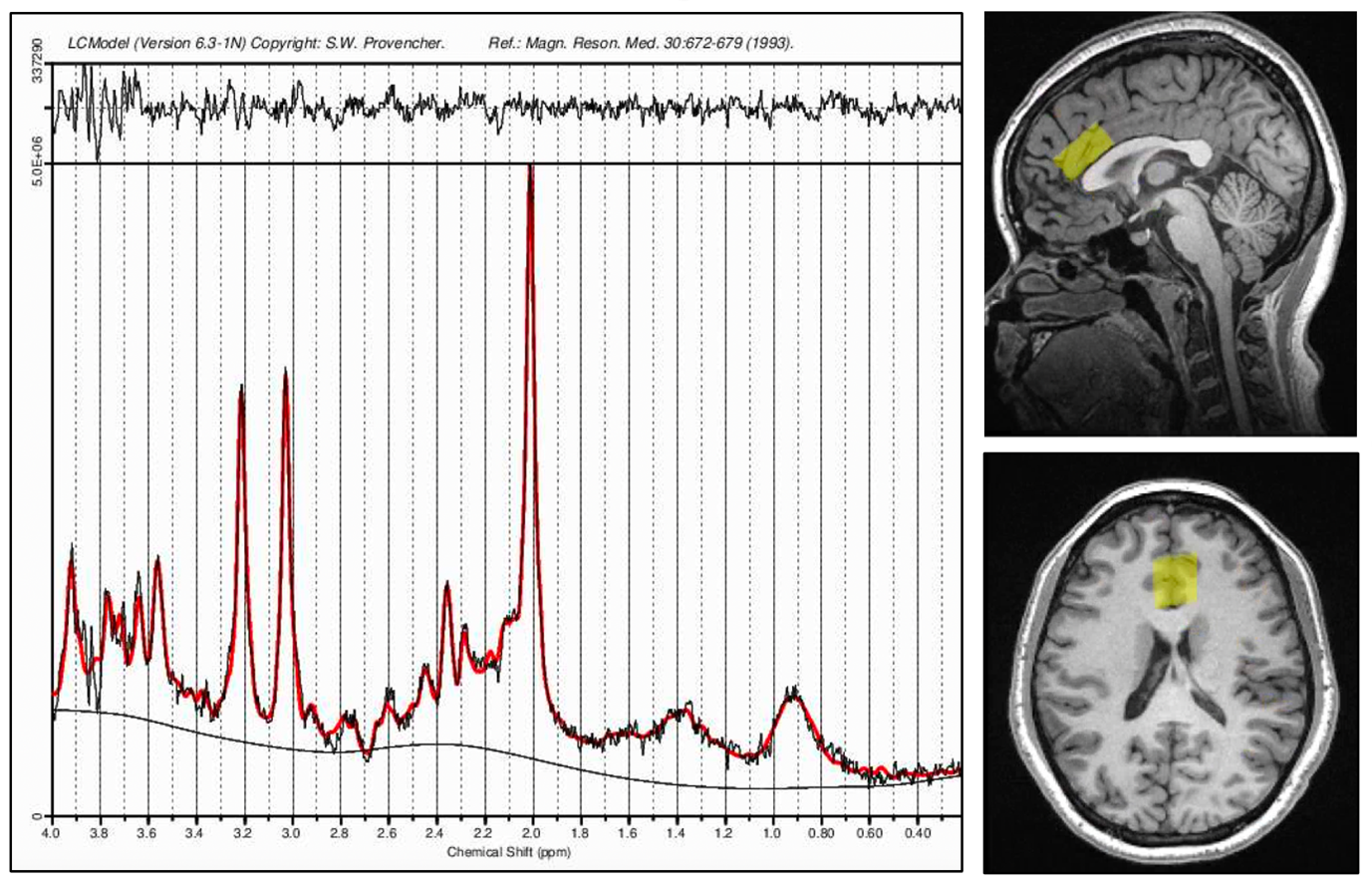


**Supplementary Figure 1:** Voxel placement in the ACC with example spectrum

**Principal Component Analysis**


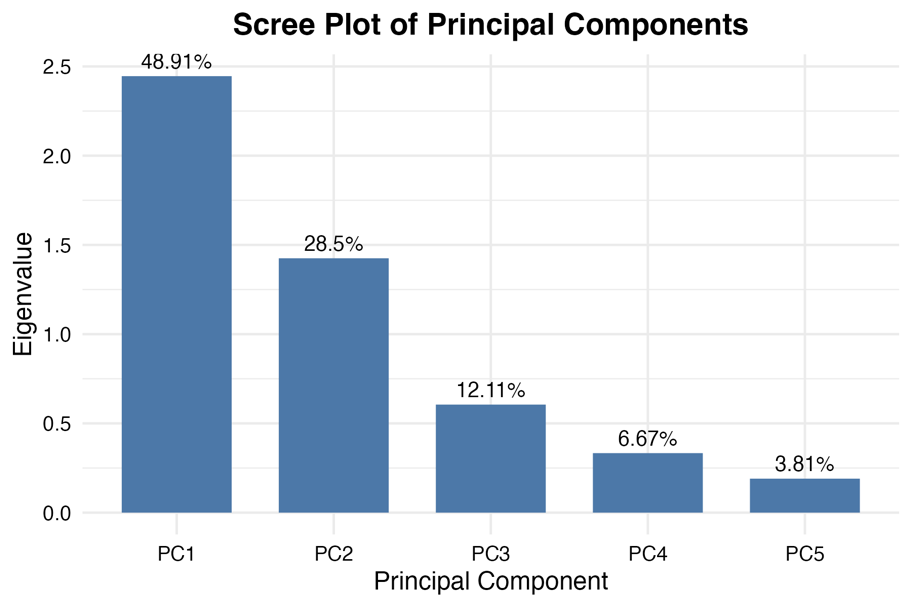


**Supplementary Figure 2:** Scree plot displaying the eigenvalues and the proportion of variance explained by each principal component. Arrows indicate the components that were retained and used in subsequent analyses.

|  | PC1 | PC2 |
| --- | --- | --- |
| Complex I | 0.501 | 0.372 |
| Complex II | 0.559 | -0.011 |
| Complex III | 0.362 | -0.580 |
| Complex IV | 0.551 | 0.101 |
| Complex V | -0.037 | 0.718 |

**Supplementary Table 1:** Factor loadings of mitochondrial complexes I–V onto the retained principal components from the PCA.

|  | | Sum Sq | DF | F value | P value |
| --- | --- | --- | --- | --- | --- |
| Glutamate | Group | 0.30 | 1 | 0.134 | 0.716 |
|  | SNR | 1.74 | 1 | 0.788 | 0.379 |
|  | Residuals | 117.01 | 53 |  |  |
| Glx | Group | 2.06 | 1 | 0.346 | 0.559 |
|  | SNR | 45.17 | 1 | 7.593 | 0.008 |
|  | Residuals | 315.31 | 53 |  |  |
| NAA | Group | 0.09 | 1 | 0.115 | 0.736 |
|  | SNR | 0.45 | 1 | 0.575 | 0.451 |
|  | Residuals | 41.08 | 53 |  |  |
| PC1 | Group | 4.61 | 1 | 1.945 | 0.169 |
|  | SNR | 1.33 | 1 | 0.561 | 0.457 |
|  | Residuals | 123.26 | 52 |  |  |
| PC2 | Group | 1.73 | 1 | 1.203 | 0.278 |
|  | SNR | 1.41 | 1 | 0.983 | 0.326 |
|  | Residuals | 74.64 | 52 |  |  |
| Lactate | Group | 0.04 | 1 | 0.052 | 0.821 |
|  | SNR | 0.01 | 1 | 0.018 | 0.894 |
|  | Residuals | 32.40 | 40 |  |  |
| Pyruvate | Group | 0.00 | 1 | 2.151 | 0.150 |
|  | SNR | 0.00 | 1 | 1.298 | 0.261 |
|  | Residuals | 0.02 | 40 |  |  |
| LP ratio | Group | 280.33 | 1 | 2.543 | 0.119 |
|  | SNR | 187.08 | 1 | 1.697 | 0.200 |
|  | Residuals | 4409.35 | 40 |  |  |

**Supplementary Table 2.** ANCOVAs testing for group differences in ^1^H-MRS metabolites and peripheral energy measures between CHR+FEP and HC, when covarying for signal-to-noise-ratio. Bootstrapping was applied for the pyruvate analysis due to non-normality. * Indicates significance at P < 0.05.

Abbreviations: Glu: glutamate; Glx: glutamate+glutamine; LP ratio: lactate-to-pyruvate ratio; NAA: N-acetylaspartate plus N-acetylaspartyl glutamate; PC1: Principal Component 1 of mitochondrial complex activity; PC2: Principal Component 2 of mitochondrial complex activity.

|  | | Sum Sq | DF | F value | P value |
| --- | --- | --- | --- | --- | --- |
| Glutamate | Group | 0.40 | 1 | 0.183 | 0.671 |
|  | Tobacco | 0.60 | 1 | 0.282 | 0.598 |
|  | Residuals | 118.10 | 53 |  |  |
| Glx | Group | 0.00 | 1 | 0.000 | 0.990 |
|  | Tobacco | 2.00 | 1 | 0.297 | 0.588 |
|  | Residuals | 358.50 | 53 |  |  |
| NAA | Group | 0.00 | 1 | 0.022 | 0.883 |
|  | Tobacco | 3.10 | 1 | 4.254 | 0.044 |
|  | Residuals | 38.40 | 53 |  |  |
| PC1 | Group | 8.80 | 1 | 3.712 | 0.060 |
|  | Tobacco | 1.35 | 1 | 0.569 | 0.454 |
|  | Residuals | 123.24 | 52 |  |  |
| PC2 | Group | 0.54 | 1 | 0.372 | 0.545 |
|  | Tobacco | 0.22 | 1 | 0.151 | 0.700 |
|  | Residuals | 75.83 | 52 |  |  |
| Lactate | Group | 0.43 | 1 | 0.565 | 0.457 |
|  | Tobacco | 1.89 | 1 | 2.474 | 0.124 |
|  | Residuals | 30.53 | 40 |  |  |
| Pyruvate | Group | 0.00 | 1 | 4.296 | **0.045*** |
|  | Tobacco | 0.00 | 1 | 6.406 | **0.015*** |
|  | Residuals | 0.02 | 40 |  |  |
| LP ratio | Group | 308.70 | 1 | 2.816 | 0.101 |
|  | Tobacco | 210.90 | 1 | 1.923 | 0.173 |
|  | Residuals | 4385.60 | 40 |  |  |

**Supplementary Table 3.** ANCOVAs testing for group differences in ^1^H-MRS metabolites and peripheral energy measures between CHR+FEP and HC, when covarying for tobacco use. Bootstrapping was applied for the pyruvate analysis due to non-normality. * Indicates significance at P < 0.05.

Abbreviations: Glu: glutamate; Glx: glutamate+glutamine; LP ratio: lactate-to-pyruvate ratio; NAA: N-acetylaspartate plus N-acetylaspartyl glutamate; PC1: Principal Component 1 of mitochondrial complex activity; PC2: Principal Component 2 of mitochondrial complex activity.

|  | | Estimate | SE | T | P value |
| --- | --- | --- | --- | --- | --- |
| **Glutamate** | | | | | |
| PC1 | Complex_PC1 | 0.180 | 0.175 | 1.028 | 0.309 |
|  | Group | -0.176 | 0.441 | -0.399 | 0.692 |
|  | PC1 * Group | -0.018 | 0.273 | -0.066 | 0.948 |
| PC2 | Complex_PC2 | 0.310 | 0.200 | 1.550 | 0.127 |
|  | Group | -0.368 | 0.425 | -0.866 | 0.391 |
|  | PC2 * Group | -0.125 | 0.380 | -0.328 | 0.744 |
| Lactate | Lactate | -0.132 | 0.342 | -0.387 | 0.701 |
|  | Group | -0.442 | 1.577 | -0.280 | 0.781 |
|  | Lactate * Group | 0.135 | 0.547 | 0.247 | 0.806 |
| Pyruvate | Pyruvate | 1.269 | 18.874 | 0.067 | 0.947 |
|  | Group | 0.942 | 1.568 | 0.601 | 0.552 |
|  | Pyruvate * Group | -11.786 | 21.839 | -0.540 | 0.592 |
| LP ratio | LP ratio | 0.021 | 0.028 | 0.750 | 0.458 |
|  | Group | 0.975 | 1.962 | 0.497 | 0.622 |
|  | LP ratio * Group | -0.026 | 0.046 | -0.576 | 0.568 |
| **Glx** | | | | | |
| PC1 | Complex_PC1 | 0.632 | 0.293 | 2.155 | **0.036*** |
|  | Group | -0.025 | 0.737 | -0.033 | 0.974 |
|  | PC1 * Group | -0.419 | 0.457 | -0.918 | 0.363 |
| PC2 | Complex_PC2 | 0.738 | 0.332 | 2.223 | **0.031*** |
|  | Group | -0.512 | 0.705 | -0.727 | 0.471 |
|  | PC2 * Group | 0.061 | 0.630 | 0.097 | 0.923 |
| Lactate | Lactate | -0.460 | 0.553 | -0.831 | 0.411 |
|  | Group | 0.095 | 2.554 | 0.037 | 0.971 |
|  | Lactate*Group | -0.042 | 0.885 | -0.047 | 0.962 |
| Pyruvate | Pyruvate | -27.697 | 30.409 | -0.911 | 0.368 |
|  | Group | -0.118 | 2.527 | -0.047 | 0.963 |
|  | Pyruvate * Group | 4.969 | 35.184 | 0.141 | 0.888 |
| LP ratio | LP ratio | 0.025 | 0.046 | 0.535 | 0.596 |
|  | Group | 1.200 | 3.233 | 0.371 | 0.713 |
|  | LP ratio * Group | -0.030 | 0.076 | -0.400 | 0.691 |
| **NAA** | | | | | |
| PC1 | Complex_PC1 | -0.038 | 0.104 | -0.362 | 0.718 |
|  | Group | -0.186 | 0.262 | -0.710 | 0.481 |
|  | PC1 * Group | 0.075 | 0.162 | 0.463 | 0.645 |
| PC2 | Complex_PC2 | 0.146 | 0.118 | 1.233 | 0.223 |
|  | Group | -0.188 | 0.251 | -0.750 | 0.457 |
|  | PC2 * Group | -0.260 | 0.224 | -1.158 | 0.252 |
| Lactate | Lactate | -0.056 | 0.205 | -0.274 | 0.785 |
|  | Group | 0.140 | 0.946 | 0.148 | 0.883 |
|  | Lactate*Group | -0.045 | 0.328 | -0.136 | 0.892 |
| Pyruvate | Pyruvate | -9.497 | 11.274 | -0.842 | 0.405 |
|  | Group | -0.236 | 0.937 | -0.252 | 0.802 |
|  | Pyruvate * Group | 4.052 | 13.045 | 0.311 | 0.758 |
| LP ratio | LP ratio | 0.016 | 0.017 | 0.952 | 0.347 |
|  | Group | 0.333 | 1.172 | 0.284 | 0.778 |
|  | LP ratio * Group | -0.009 | 0.027 | -0.323 | 0.748 |

**Supplementary Table 4.** General linear models of associations between ^1^H-MRS metabolites and peripheral energy measures, including a group * metabolite interaction term. Bootstrapping was applied to the pyruvate analysis due to non-normality. * Indicates significance at P < 0.05.

Abbreviations: Glu: glutamate; Glx: glutamate+glutamine; LP ratio: lactate-to-pyruvate ratio; NAA: N-acetylaspartate plus N-acetylaspartyl glutamate; PC1: Principal Component 1 of mitochondrial complex activity; PC2: Principal Component 2 of mitochondrial complex activity.

|  | | Estimate | SE | statistic | P value |
| --- | --- | --- | --- | --- | --- |
| **Glutamate** | | | | | |
| PC1 | Complex PC1 | 0.196 | 0.178 | 1.104 | 0.275 |
|  | Group | -0.066 | 0.472 | -0.139 | 0.890 |
|  | Tobacco | 0.380 | 0.566 | 0.671 | 0.506 |
|  | PC1 * Group | -0.034 | 0.275 | -0.124 | 0.902 |
| PC2 | Complex PC2 | 0.318 | 0.202 | 1.577 | 0.121 |
|  | Group | -0.271 | 0.454 | -0.597 | 0.553 |
|  | Tobacco | 0.350 | 0.557 | 0.629 | 0.532 |
|  | PC2 * Group | -0.133 | 0.383 | -0.347 | 0.730 |
| Lactate | Lactate | -0.099 | 0.363 | -0.273 | 0.786 |
|  | Group | -0.292 | 1.676 | -0.174 | 0.862 |
|  | Tobacco | 0.189 | 0.648 | 0.292 | 0.772 |
|  | Lactate * Group | 0.102 | 0.564 | 0.181 | 0.857 |
| Pyruvate | Pyruvate | 1.269 | 19.121 | 0.066 | 0.947 |
|  | Group | 0.947 | 1.701 | 0.556 | 0.581 |
|  | Tobacco | 0.005 | 0.677 | 0.007 | 0.994 |
|  | Pyruvate * Group | -11.825 | 22.750 | -0.520 | 0.606 |
| LP ratio | LP ratio | 0.019 | 0.030 | 0.657 | 0.515 |
|  | Group | 0.953 | 1.989 | 0.479 | 0.635 |
|  | Tobacco | 0.131 | 0.637 | 0.205 | 0.839 |
|  | LP ratio * Group | -0.025 | 0.047 | -0.525 | 0.602 |
| **Glx** | | | | | |
| PC1 | Complex PC1 | 0.666 | 0.297 | 2.247 | 0.029 |
|  | Group | 0.211 | 0.788 | 0.268 | 0.790 |
|  | Tobacco | 0.815 | 0.944 | 0.863 | 0.392 |
|  | PC1 * Group | -0.454 | 0.459 | -0.988 | 0.328 |
| PC2 | Complex PC2 | 0.753 | 0.334 | 2.253 | 0.029 |
|  | Group | -0.331 | 0.753 | -0.440 | 0.662 |
|  | Tobacco | 0.658 | 0.922 | 0.714 | 0.479 |
|  | PC2 * Group | 0.046 | 0.634 | 0.073 | 0.942 |
| Lactate | Lactate | -0.468 | 0.590 | -0.794 | 0.432 |
|  | Group | 0.057 | 2.718 | 0.021 | 0.983 |
|  | Tobacco | -0.047 | 1.052 | -0.045 | 0.964 |
|  | Lactate * Group | -0.034 | 0.915 | -0.037 | 0.971 |
| Pyruvate | Pyruvate | -27.697 | 30.752 | -0.901 | 0.373 |
|  | Group | 0.241 | 2.736 | 0.088 | 0.930 |
|  | Tobacco | 0.400 | 1.089 | 0.367 | 0.716 |
|  | Pyruvate * Group | 1.842 | 36.587 | 0.050 | 0.960 |
| LP ratio | LP ratio | 0.024 | 0.049 | 0.489 | 0.627 |
|  | Group | 1.187 | 3.280 | 0.362 | 0.719 |
|  | Tobacco | 0.071 | 1.051 | 0.068 | 0.946 |
|  | LP ratio * Group | -0.029 | 0.078 | -0.378 | 0.708 |
| **NAA** | | | | | |
| PC1 | Complex PC1 | -0.010 | 0.102 | -0.098 | 0.922 |
|  | Group | 0.002 | 0.271 | 0.007 | 0.994 |
|  | Tobacco | 0.649 | 0.325 | 1.999 | 0.051 |
|  | PC1 * Group | 0.047 | 0.158 | 0.300 | 0.766 |
| PC2 | Complex PC2 | 0.161 | 0.114 | 1.410 | 0.165 |
|  | Group | 0.000 | 0.257 | -0.001 | 1.000 |
|  | Tobacco | 0.682 | 0.315 | 2.162 | 0.035 |
|  | PC2 * Group | -0.275 | 0.217 | -1.271 | 0.210 |
| Lactate | Lactate | 0.055 | 0.211 | 0.260 | 0.796 |
|  | Group | 0.646 | 0.971 | 0.666 | 0.510 |
|  | Tobacco | 0.640 | 0.376 | 1.705 | 0.096 |
|  | Lactate * Group | -0.156 | 0.327 | -0.476 | 0.637 |
| Pyruvate | Pyruvate | -9.497 | 11.097 | -0.856 | 0.397 |
|  | Group | -0.767 | 0.987 | -0.777 | 0.442 |
|  | Tobacco | -0.591 | 0.393 | -1.503 | 0.141 |
|  | Pyruvate * Group | 8.675 | 13.202 | 0.657 | 0.515 |
| LP ratio | LP ratio | 0.009 | 0.017 | 0.523 | 0.604 |
|  | Group | 0.235 | 1.156 | 0.203 | 0.840 |
|  | Tobacco | 0.557 | 0.370 | 1.506 | 0.140 |
|  | LP ratio * Group | -0.002 | 0.027 | -0.067 | 0.947 |

**Supplementary Table 5.** General linear models testing associations between ^1^H-MRS metabolites and peripheral energy measures, including a group * metabolite interaction term, and covarying for tobacco use. Bootstrapping was applied to the pyruvate analysis due to non-normality.

Abbreviations: Glu: glutamate; Glx: glutamate+glutamine; LP ratio: lactate-to-pyruvate ratio; NAA: N-acetylaspartate plus N-acetylaspartyl glutamate; PC1: Principal Component 1 of mitochondrial complex activity; PC2: Principal Component 2 of mitochondrial complex activity.

| **WCST - Mean Categories** | | | | |
| --- | --- | --- | --- | --- |
|  | N | Estimate | Statistic | P value |
| Glutamate | 47 | -0.177 | -1.209 | 0.233 |
| Glx | 47 | -0.139 | -0.941 | 0.352 |
| NAA | 47 | -0.053 | -0.356 | 0.723 |
| PC1 | 45 | -0.148 | -0.993 | 0.326 |
| PC2 | 45 | -0.157 | -1.053 | 0.298 |
| Lactate | 36 | -0.168 | -0.991 | 0.328 |
| Pyruvate | 36 | -0.147 | -0.869 | 0.391 |
| LP ratio | 36 | 0.089 | 0.522 | 0.605 |

**Supplementary Table 6.**Bivariate correlations of associations between ^1^H-MRS metabolites and peripheral energy measures with the number of categories completed on the Wisconsin Card Sorting Test (WCST) across all participants.

Abbreviations: Glu: glutamate; Glx: glutamate+glutamine; LP ratio: lactate-to-pyruvate ratio; NAA: N-acetylaspartate plus N-acetylaspartyl glutamate; PC1: Principal Component 1 of mitochondrial complex activity; PC2: Principal Component 2 of mitochondrial complex activity.

| **WCST – Perseverative errors** | | | | |
| --- | --- | --- | --- | --- |
|  | N | Estimate | Statistic | P value |
| Glutamate | 47 | -0.206 | -1.409 | 0.166 |
| Glx | 47 | -0.168 | -1.146 | 0.258 |
| NAA | 47 | -0.171 | -1.165 | 0.250 |
| PC1 | 45 | -0.061 | -0.407 | 0.686 |
| PC2 | 45 | -0.165 | -1.110 | 0.273 |
| Lactate | 36 | -0.046 | -0.268 | 0.790 |
| Pyruvate | 36 | -0.105 | -0.614 | 0.543 |
| LP ratio | 36 | 0.094 | 0.548 | 0.587 |

**Supplementary Table 7.**Bivariate correlations of associations between ^1^H-MRS metabolites and peripheral energy measures with the number of perseverative errors on the Wisconsin Card Sorting Test (WCST) across the full sample.

Abbreviations: Glu: glutamate; Glx: glutamate+glutamine; LP ratio: lactate-to-pyruvate ratio; NAA: N-acetylaspartate plus N-acetylaspartyl glutamate; PC1: Principal Component 1 of mitochondrial complex activity; PC2: Principal Component 2 of mitochondrial complex activity.

| **Positive Symptom Severity** | | | | |
| --- | --- | --- | --- | --- |
|  | N | Estimate | Statistic | P value |
| Glutamate | 36 | -0.129 | -0.760 | 0.452 |
| Glx | 36 | -0.135 | -0.794 | 0.433 |
| NAA | 36 | 0.011 | 0.065 | 0.949 |
| PC1 | 35 | -0.174 | -1.030 | 0.310 |
| PC2 | 35 | 0.019 | 0.109 | 0.914 |
| Lactate | 29 | 0.042 | 0.218 | 0.829 |
| Pyruvate | 29 | -0.227 | -1.212 | 0.236 |
| LP ratio | 29 | 0.386 | 2.175 | 0.039 |

**Supplementary Table 8.**Bivariate correlations of associations between ^1^H-MRS metabolites and peripheral energy measures with positive symptom severity in the CHR+FEP group.

Abbreviations: Glu: glutamate; Glx: glutamate+glutamine; LP ratio: lactate-to-pyruvate ratio; NAA: N-acetylaspartate plus N-acetylaspartyl glutamate; PC1: Principal Component 1 of mitochondrial complex activity; PC2: Principal Component 2 of mitochondrial complex activity.
